# Protease Activated Receptors 1 and 2 Correlate Differently with Breast Cancer Aggressiveness Depending on Tumor ER Status

**DOI:** 10.1371/journal.pone.0134932

**Published:** 2015-08-05

**Authors:** Jon Lidfeldt, Pär-Ola Bendahl, Carina Forsare, Per Malmström, Mårten Fernö, Mattias Belting

**Affiliations:** 1 Department of Clinical Sciences, Section of Oncology and Pathology, Lund University, Lund, Sweden; 2 Department of Oncology, Skåne University Hospital, Lund, Sweden; University of North Carolina School of Medicine, UNITED STATES

## Abstract

Experimental models implicate protease activated receptors (PARs) as important sensors of the proteolytic tumor microenvironment during breast cancer development. However, the role of the major PARs, PAR-1 and PAR-2, in human breast tumors remains to be elucidated. Here, we have investigated how PAR-1 and PAR-2 protein expression correlate with established clinicopathological variables and patient outcome in a well-characterized cohort of 221 breast cancer patients. Univariable and multivariable hazard ratios (HR) were estimated by the Cox proportional hazards model, distant disease-free survival (DDFS) and overall survival by the Kaplan–Meier method, and survival in different strata was determined by the log-rank test. Associations between PARs and clinicopathological variables were analyzed using Pearson’s χ^2^-test. We find that PAR-2 associates with DDFS (HR = 3.1, P = 0.003), whereas no such association was found with PAR-1 (HR = 1.2, P = 0.6). Interestingly, the effect of PAR-2 was confined to the ER-positive sub-group (HR = 5.5, P = 0.003 *vs*. HR = 1.2 in ER-negative; P = 0.045 for differential effect), and PAR-2 was an independent prognostic factor specifically in ER-positive tumors (HR = 3.9, P = 0.045). On the contrary, PAR-1 correlated with worse prognosis specifically in the ER-negative group (HR = 2.6, P = 0.069 *vs*. HR = 0.5, P = 0.19 in ER-positive; P = 0.026 for differential effect). This study provides novel insight into the respective roles of PAR-1 and PAR-2 in human breast cancer and suggests a hitherto unknown association between PARs and ER signaling that warrants further investigation.

## Introduction

Breast cancer is a heterogeneous disease with a substantial variation in aggressiveness and prognosis [1]. Proteases of the tumor microenvironment have emerged as important regulators of cancer cell invasiveness and metastatic capacity through stroma remodeling and increased angiogenesis. Moreover, proteolytic activity in the extracellular environment through *e*.*g*. matrix metalloproteinases and coagulation proteases may directly cleave and activate a unique class of G protein-coupled protease activated receptors (PARs), most importantly PAR-1 and PAR-2. PARs are known to be expressed at variable levels by malignant as well as tumor stromal cells, and have been implicated as regulators of tumor vascularization and metastasis [2–4]. Collectively, experimental studies point at a major role of PAR-2 in breast tumor development, whereas the role of PAR-1 is less clear. PAR-1 deficiency had no effect on tumor development and metastasis in a transgenic model of spontaneous breast cancer, whereas PAR-2 knock-out mice displayed delayed tumor formation and decreased lung metastases [5]. Moreover, blocking antibodies directed at PAR-2 but not PAR-1 were shown to attenuate tumor growth and metastasis in a breast xenograft model [6], and shRNA-mediated silencing of PAR-2 but not PAR-1 mRNA in breast cancer cells showed a specific role of PAR-2 in promoting the malignant cell phenotype [7]. In one study, PAR-1 activation was even found to inhibit breast cancer cell migration [8]. Several other studies, however, suggest that PAR-1 has an important role in the progression of breast cancer [9–12].

Interestingly, there may be a more complex interrelationship between the PARs, as suggested by experimental studies showing that PAR-1 cleavage can transactivate PAR-2 [13, 14]. At the molecular level, it has been demonstrated that PAR-1 and PAR-2 can heterodimerize and co-traffic during internalization [15]. PAR-1 and PAR-2 heterodimerization may have functional importance as suggested from studies showing that PAR-2 expression and co-signaling are necessary for PAR-1-induced hyperplasia of vessel intima [16]. More recently, it was proposed that the presence of PAR-2 is required for PAR-1-induced signaling events associated with breast tumor development. The same study suggested that this is not a reciprocal mechanism since PAR-2-dependent stimulatory effects in breast cancer cells were intact even in the absence of PAR-1 [17].

Together, previous investigations in experimental systems thus implicate that PAR-1 and PAR-2 may act either independently or together as a functional unit to regulate breast tumor development. However, the role of PARs and the interrelationship of PAR-1 and PAR-2 in human breast cancer remain poorly defined [18, 19]. In the present study, we used a well-characterized cohort of premenopausal patients with lymph node-negative breast cancer to investigate the role of PARs with a specific focus on whether the prognostic value of PARs in breast cancer differs depending on tumor ER status.

## Materials and Methods

### Patient characteristics

The patient population encompassed 237 premenopausal patients with lymph node-negative breast cancer from a prospective study in southern Sweden during 1991 to 1994 [20]. Our studies were performed according to the recommendations of "REMARK" guidelines [21]. The study was approved by the ethics committee of Lund University Hospital. All participants provided their written consent to participate in the study. From the initial 237 patients, 221 tumors samples were scored for PAR-1 and PAR-2 expression. In 14 cases, paraffin blocks were not retrieved from the pathology departments, and in the remaining two cases individual tumor sections were either lost in the preparation of tissue microarray (TMA) or judged non-evaluable due to insufficient number of malignant cells or insufficient malignant tissue. Primary surgical treatment, postoperative radiation, and adjuvant systemic treatment have been described in detail earlier [20] (see also, **Table 1**). The median follow-up for the end-point distant metastasis was 10.9 years for the 168 patients who were alive and free from distant metastases at the latest review of the patients’ records. Results for the first 5 and 10 years are presented, as indicated. Histological grading of tumors was performed according to Elston and Ellis [22]. All tumor specimens were re-evaluated by seven experienced pathologists without knowledge of patient history [20]. Patient and tumor characteristics are summarized in **Table 1**.

**Table 1:** Patient and breast tumor characteristics in 221 premenopausal patients with lymph-node negative breast cancer.

Variable		
	**Median**	**Range**
**Age,** years	47	30–57
**Tumor size,** mm	15	5–30
**Histological grade**	**No. of Patients**	**% of total**
1	67	31
2	79	37
3	70	32
Not determined	5	
**Adjuvant therapy**		
Chemotherapy	21	9.5
Tamoxifen	7	3
Ovarian ablation	1	0.5
None	192	87
**5 year follow-up**	**%**	**95% CI**
Cumulative distant recurrence	15.4	11.0–20.5
Cumulative breast cancer mortality	7.7	4.7–11.7
Cumulative mortality	8.2	5.0–12.2
**10 year follow-up**		
Cumulative breast cancer mortality	19.5	14.6–25.0
Cumulative mortality	20.4	15.4–26.0

### Tumor tissue microarray

A TMA was obtained from the paraffin embedded tumor specimens. Two 0.6 mm core biopsies were taken from representative areas of each tumor, and transferred into a new paraffin block using a manual arrayer (Beecher Instruments, MD, USA). Sections of 4 μm were stained with haematoxylin and eosin B.

### Immunohistochemistry

ER, PR, HER-2, VEGF-A, and Ki-67 analyses were performed as described earlier [23, 24]. Seven tumors were non-evaluable for HER-2 due to insufficient tumor material or fixation artifacts. All patients with amplified tumors according to FISH analyses, and all with Herceptest 3+ where FISH analysis could not be evaluated, were considered HER-2 positive. Expression of PAR-1 and PAR-2 were determined using the DAKO Envision kit K 5007 (an indirect polymer reinforcement technique) in a TechMate 500Plus, (DAKO, Copenhagen, Denmark). Antigen retrieval was performed by treatment in a microwave oven in target retrieval solutions pH 6 (PAR-2) or pH 9 (PAR-1). Sections were incubated with the primary antibody for 30 min (PAR-1) or 2 h (PAR-2). Antibodies used were mouse anti-human PAR-1 (sc-13503, Santa Cruz; 1:150 dilution) and mouse anti-human PAR-2 (sc-13504, Santa Cruz; 1:100 dilution). Diaminobenzidine (DAB) was used for visualization. Negative control sections were performed by omitting the primary antibody in each staining batch, and sections were counter-stained with haematoxylin. Slides were reviewed by two independent examiners (J.L. and M.B.) without knowledge of clinical and pathological information. A homogenous staining of tumors for PAR-1 and PAR-2 was observed; therefore, a scoring system based on percentage of positive cells was not further considered. Scoring of PAR-1 and PAR-2 was performed semi-quantitatively according to staining intensity on a scale as follows: 0 = total negative slide, 1 = weak, 2 = moderate, 3 = strong and 4 = very strong intensity (**S1 Fig**). Magnifications ranging from 4x to 40x were used during scoring.

### Statistical analyses

Distant disease-free survival (DDFS) was primary end-point and breast cancer mortality (BCM) secondary end-point. The Cox proportional hazards model was used for estimation of univariable and multivariable hazard ratios (HR). Proportional hazards (PH) assumptions were checked both graphically and by Schoenfeld’s test [25]. Deviations from PH were observed, motivating truncation of follow-up for DDFS at 5 years. The deviations from PH were less in analyses of BCM. Hence, also 10 years of follow-up could be used for this endpoint. Estimated HRs should, however, be interpreted as average effects over time. The Kaplan–Meier method was used to estimate DDFS and overall survival (OS) whereas a slightly modified method [26] accounting for competing events, *i*.*e*. deaths from other causes, was used to estimate BCM. The log-rank test was used to compare survival in different strata. All factors were used as dichotomous covariates in the statistical analyses except for histological grade (three groups) and age which was analyzed as a continuous variable. Cut-off values were defined before statistical analyses. For the established prognostic factors (ER, PR, HER-2) standard cut-off values were used, and were the same as in previously published patient series [20]. Associations between the dichotomized PAR variables and other dichotomized variables were analyzed using Pearson’s χ^2^-test. The trend version of the test was used for histological grade. All P-values corresponded to two-sided tests. When referring to a statistically significant effect, we mean a P-value below the threshold 0.05, but the P-value should rather be interpreted as level of evidence against the null hypothesis. The statistical calculations were performed using Stata Version 13.1 (StataCorp LP, 2014, College Station, TX, USA).

## Results

### Patient and tumor characteristics

Detailed characteristics of the patients and tumors are presented in **Table 1**; notably, the vast majority (87%) of patients in this cohort received no adjuvant systemic therapy, which makes it suitable to more specifically assess the prognostic value of biomarkers. Among more established clinicopathological variables, high PAR-1 was only significantly associated with high Ki-67 (>20%), whereas high PAR-2 was significantly associated with younger age (<50), larger tumor size (>20 mm), high histological grade, high Ki-67 and ER- and PR-negativity (**Table 2**). Further, PAR-1 and PAR-2 expressions were shown to be positively associated (P = 0.019). Whereas PAR-2 was specifically found in malignant cells, high PAR-1 expression was found in malignant as well as neighbouring stromal cells (**S1 Fig**).

**Table 2:** Associations between other prognostic factors and PAR-1 and PAR-2, respectively.

			PAR-1					PAR-2				
			Low		High			Low		High		
Variable	n	%	n	%	n	%	P-value	n	%	n	%	P-value
**All**	221	100	112	51	109	49		119	54	102	46	
**Age**							0.6					0.046
<50 years	166	75	86	52	80	48		83	50	83	50	
≥50 years	55	25	26	47	29	53		36	65	19	35	
**Tumor size**							0.7					0.001
<20 mm	165	75	85	52	80	48		100	61	65	39	
≥20 mm	56	25	27	48	29	52		19	34	37	66	
**Histological grade**	216						0.3*					<0.001*
1	67	31	36	54	31	46		47	70	20	30	
2	79	37	42	53	37	47		48	61	31	39	
3	70	32	31	44	39	56		20	29	50	71	
Not determined	5											
**Ki-67**	197						0.007					<0.001
≤20%	135	69	76	56	59	44		86	64	49	36	
>20%	62	31	22	35	40	65		18	29	44	71	
Not determined	24											
**HER-2**	207						0.2					0.3
Neg	184	89	98	53	86	47		103	56	81	44	
Pos	23	11	9	39	14	61		10	43	13	57	
Not determined	14											
**ER**							0.4					<0.001
Neg	75	34	35	47	40	53		28	37	47	63	
Pos	146	66	77	53	69	47		91	62	55	38	
**PR**							0.10					<0.001
Neg	62	28	26	42	36	58		18	29	44	71	
Pos	159	72	86	54	73	46		101	64	58	36	

*Chi2-test; the trend version for histological grade.

### Association of PAR-1 and PAR-2 expression to patient outcome depends on ER status

**Fig 1:**
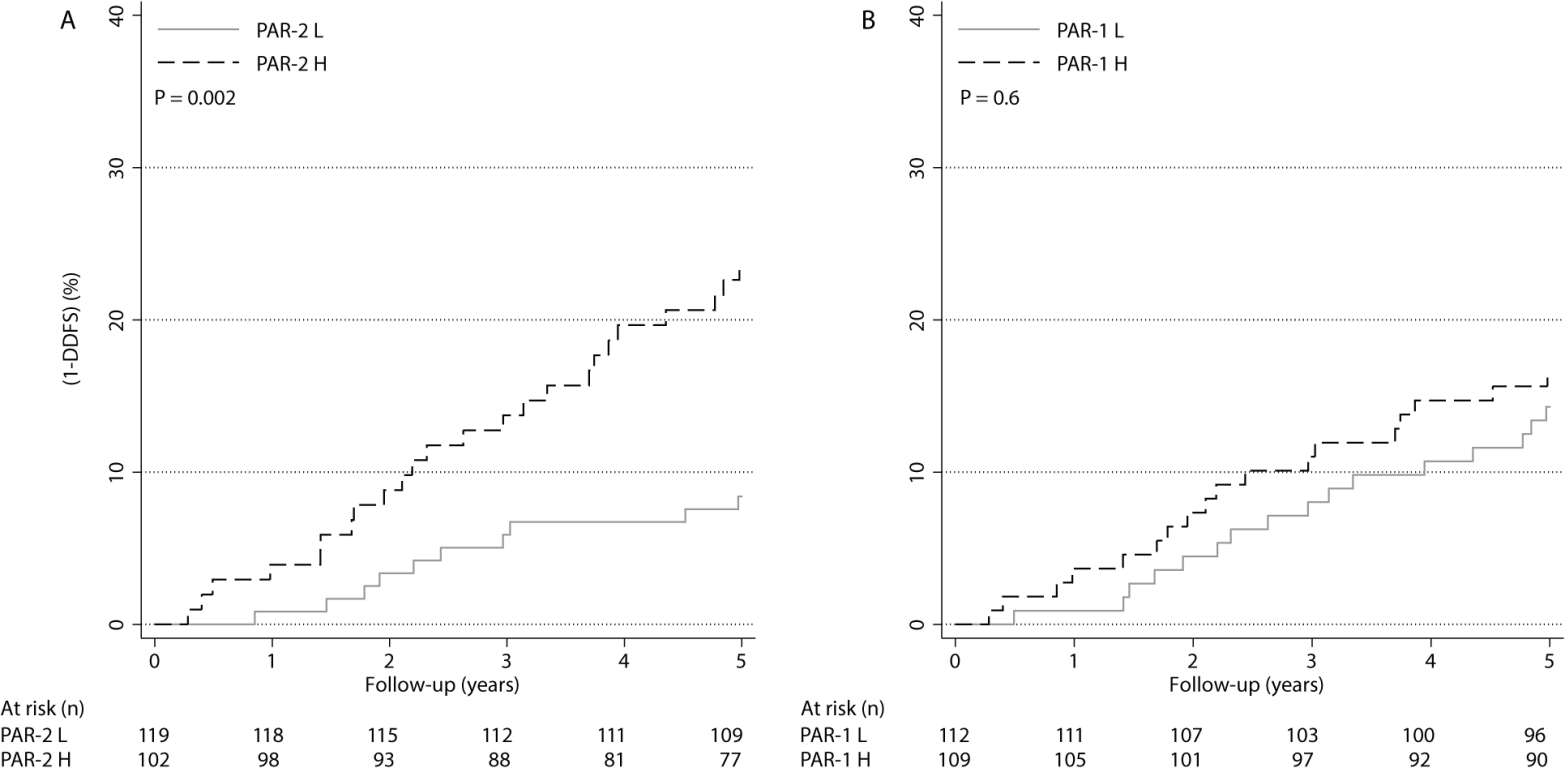
One minus distant disease-free survival (DDFS) stratified by PARs. Prognosis in relation to PAR-2 (a) and PAR-1 (b) status in the entire cohort.

In univariable analysis, PAR-2 was a prognostic factor for DDFS (HR: 3.1, 95% CI: 1.5–6.4, P = 0.003), whereas PAR-1 showed no such correlation (HR: 1.2, 95% CI: 0.6–2.3, P = 0.6) (**Table 3**). The DDFS was 92 and 76% in low- and high-PAR-2 expressing groups, respectively (**Fig 1A**). In univariable analysis of BCM during the first 10 years after diagnosis, PAR-2 remained as a significant prognostic factor (HR: 2.4, 95% CI: 1.3–4.5, P = 0.006) (**Table 3**). The corresponding cumulative BCM (95% CI) was 13% (7–19%) and 27% (19–36%) in low- and high-PAR-2 expressing groups, respectively (**S2A Fig**). Overall, HER-2, PAR-2, Ki-67, histological grade, ER age and PR were significant prognostic factors in univariable analysis of DDFS whereas tumor size and PAR-1 were not significantly associated to DDFS (**Table 3**and **Fig 1B**).

**Table 3:** Univariable Cox regression analysis of factors for survival within 5 (DDFS) and 10 (BCM) years.

		DDFS, 5 years					BCM, 10 years				
Variable	n	HR	95% CI	P-value	n	%	HR	95% CI	P-value	n	%
**Age**											
<50 years	166	5.8	1.4–24	0.016	32	19	3.6	1.3–9.9	0.016	39	23
≥50 years	55	1.0	Reference		2	4	1.0	Reference		4	7
**Tumor size**											
<20 mm	165	1.0	Reference		22	13	1.0	Reference		28	17
≥20 mm	56	1.8	0.87–3.6	0.11	12	21	1.7	0.91–3.2	0.095	15	27
**Histological grade**				0.009*					0.004*		
1	67	1.0	Reference		5	7	1.0	Reference		10	15
2	79	1.9	0.67–5.6	0.2	11	14	0.85	0.36–2.1	0.7	10	13
3	70	4.0	1.5–11	0.007	18	26	2.6	1.2–5.4	0.013	23	33
**Ki-67**											
≤20%	135	1.0	Reference		15	11	1.0	Reference		22	16
>20%	62	2.8	1.4–5.6	0.004	17	27	2.1	1.2–4.0	0.015	19	31
**HER-2**											
Neg	184	1.0	Reference		19	10	1.0	Reference		27	15
Pos	23	6.1	2.9–13	<0.001	11	48	4.6	2.3–9.4	<0.001	11	48
**ER**											
Neg	75	2.4	1.2–4.7	0.011	18	24	2.1	1.2–3.8	0.015	21	28
Pos	146	1.0	Reference		16	11	1.0	Reference		22	15
**PR**											
Neg	62	3.0	1.5–5.8	0.001	17	27	2.7	1.5–4.8	0.001	20	32
Pos	159	1.0	Reference		17	11	1.0	Reference		23	14
**PAR-1**											
Low	112	1.0	Reference		16	14	1.0	Reference		20	18
High	109	1.2	0.61–2.3	0.6	18	17	1.2	0.67–2.2	0.5	23	21
**PAR-2**											
Low	119	1.0	Reference		10	8	1.0	Reference		15	13
High	102	3.1	1.5–6.4	0.003	24	24	2.4	1.3–4.5	0.006	28	27

*2-df likelihood ratio test.

**Fig 2:**
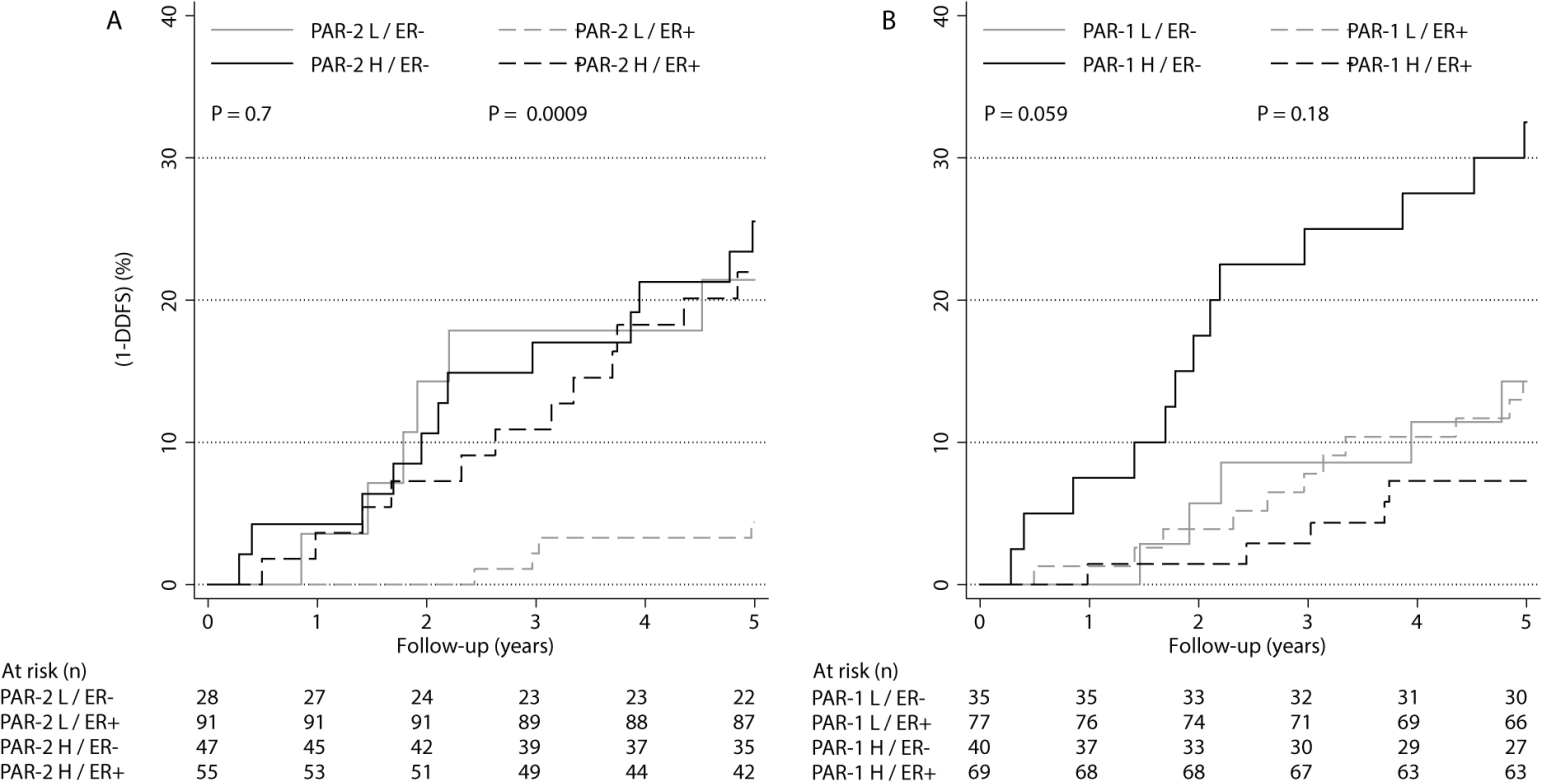
One minus distant disease-free survival (DDFS) stratified by ER in PAR-2 and PAR-1 sub-groups. Prognosis in relation to PAR-2 (a) and PAR-1 (b) status in patients with ER-positive and ER-negative tumors.

The prognostic value of the PAR variables was evaluated in subgroups defined by established prognostic factors (**Table 4**). These analyses revealed a negative effect of high PAR-2 in younger patients whose tumors were small, low grade, low Ki-67, HER-2-negative, and ER- and PR-positive. Interestingly, the most striking differential effect of high PAR-2 was found depending on ER status; the effect in the ER-positive group was HR: 5.5 (95% CI: 1.8–17, P = 0.003) compared to HR: 1.2 (95% CI: 0.4–3.2, P = 0.7) in the ER-negative group. The differential effect was found to be significant (P = 0.045) in a Cox model with a term for the interaction between the two variables and it remained significant in analysis of BCM (**Table 4**and **S3 Fig**). In ER-positive tumors, the DDFS was 96% and 78% in low- and high-PAR-2 expressing groups, respectively (**Fig 2A**). In multivariable analysis of DDFS, PAR-2 was an independent prognostic factor in the ER-positive group (HR: 3.9, 95% CI: 1.03–15.0, P = 0.045) when adjusting for age, tumor size, grade, Ki-67and HER-2 status.

**Table 4:** Univariable Cox regression analysis of PAR-1 and PAR-2 for survival in relation to other variables.

	DDFS, 5 years						BCM, 10 years					
	PAR-1 High vs Low			PAR-2 High vs Low			PAR-1 High vs Low			PAR-2 High vs Low		
Variable	HR	95% CI	P	HR	95% CI	P	HR	95% CI	P	HR	95% CI	P
**Age**												
<50 years	1.3	0.64–2.6	0.5	3.3	1.5–7.4	0.003	1.2	0.62–2.2	0.6	2.5	1.3–5.0	0.007
≥50 years	*			*			*			*		
**Tumor size**												
<20 mm	1.3	0.56–3.0	0.5	3.6	1.5–8.9	0.005	1.5	0.69–3.1	0.3	2.7	1.3–5.7	0.011
≥20 mm	1.0	0.32–3.1	1.0	1.6	0.43–5.9	0.5	0.86	0.31–2.4	0.8	1.5	0.48–4.7	0.5
**Histological grade**											
1–2	0.92	0.34–2.5	0.9	4.5	1.6–13	0.005	0.97	0.40–2.3	0.9	2.5	1.0–6.1	0.041
3	1.3	0.50–3.3	0.6	1.0	0.37–2.9	1.0	1.3	0.54–2.9	0.6	1.2	0.46–3.0	0.7
**Ki-67**												
≤20%	0.63	0.22–1.8	0.4	3.8	1.3–11	0.014	0.71	0.30–1.7	0.4	2.3	0.99–5.3	0.052
>20%	1.4	0.50–4.1	0.5	1.3	0.43–4.1	0.6	1.2	0.47–3.3	0.7	1.2	0.43–3.3	0.7
**HER-2**												
Neg	1.0	0.43–2.6	0.9	2.9	1.1–7.7	0.029	1.1	0.50–2.3	0.9	2.0	0.92–4.3	0.081
Pos	0.69	0.21–2.3	0.5	2.3	0.60–8.6	0.2	0.64	0.20–2.1	0.5	2.4	0.64–9.1	0.2
**ER**												
neg	2.6	0.93–7.3	0.069	1.2	0.45–3.2	0.7	2.0	0.82–5.0	0.13	1.5	0.60–4.0	0.4
pos	0.49	0.17–1.4	0.19	5.5	1.8–17	0.003	0.73	0.31–1.7	0.5	2.7	1.2–6.3	0.022
**PR**												
neg	1.5	0.55–4.0	0.4	0.96	0.34–2.7	0.9	1.2	0.48–2.9	0.7	1.2	0.45–3.4	0.7
pos	0.81	0.31–2.1	0.7	4.6	1.6–13	0.004	1.1	0.47–2.4	0.9	2.5	1.1–5.7	0.029

*Number of failures below 10.

In contrast, high PAR-1 had no significant effect in any of the analyzed subgroups. Interestingly, however, the strongest PAR-1 effects were observed in subgroups of ER and these effects were in opposite directions as compared with PAR-2, indicating an interaction effect on prognosis also between ER and PAR-1; high PAR-1 was found to be associated to worse prognosis in the ER-negative group (HR: 2.6, 95% CI: 0.9–7.3, P = 0.069), whereas there was no significant effect in the ER-positive group (HR: 0.5, 95% CI: 0.17–1.4, P = 0.19) (**Table 4**). This differential effect was also found to be significant (P = 0.026). In ER-negative tumors, the DDFS was 86% and 68% in low- and high-PAR-1 expressing groups, respectively (**Fig 2B**). In multivariable analysis of DDFS, however, the effect of PAR-1 in ER-negative tumors was insignificant (HR: 1.5, 95% CI: 0.42–5.7, P = 0.5).

### Interactions between ER, PAR-1, and PAR-2

**Fig 3:**
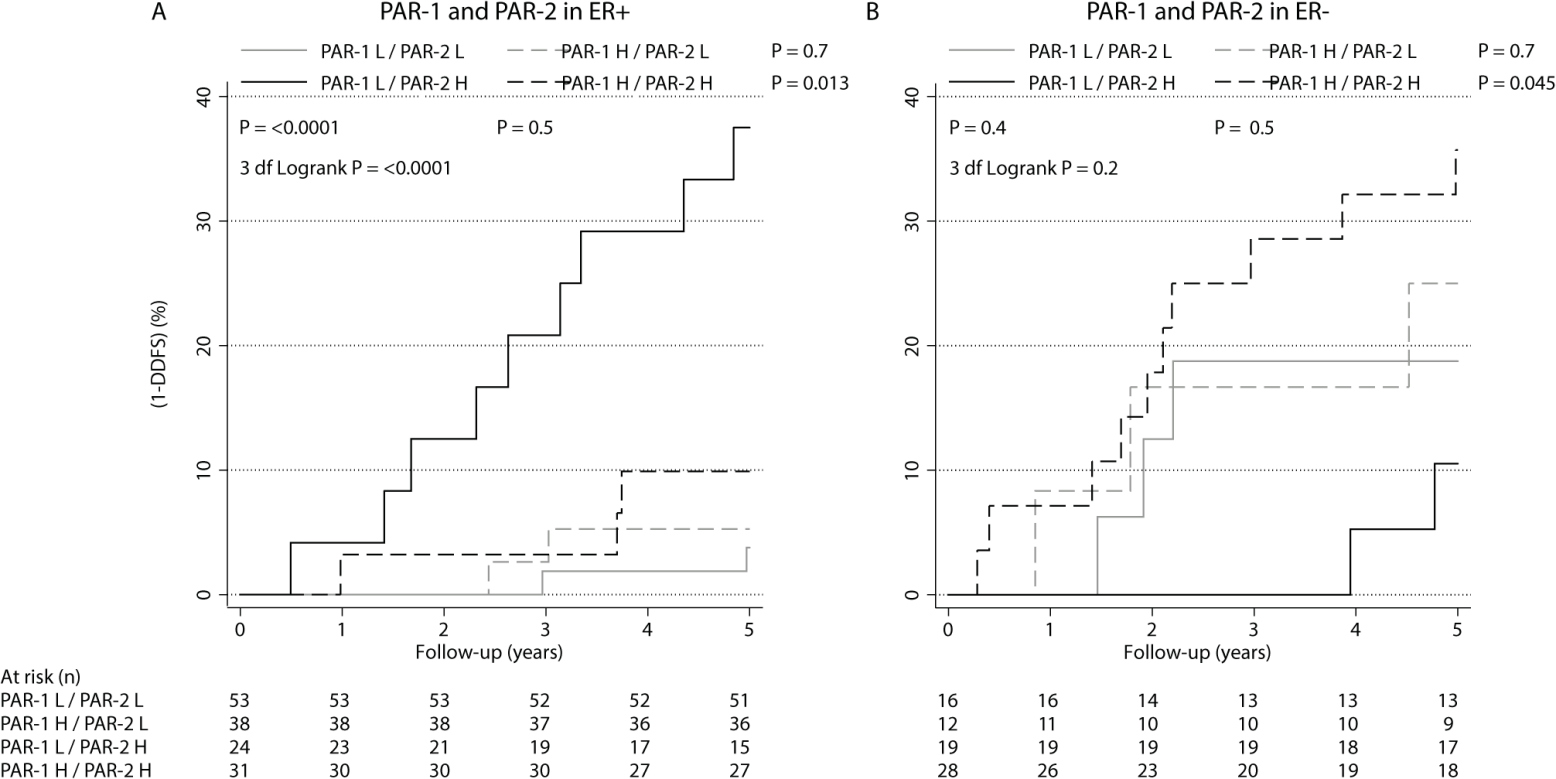
One minus distant disease-free survival (DDFS) stratified by PARs in ER-positive and ER-negative subgroups. Prognosis in relation to PAR-1 and PAR-2 status in patients with (a) ER-positive and (b) ER-negative tumors.

Previous experimental studies have established that PAR-1 and PAR-2 may heterodimerize, and co-signal during breast tumor development [13–17]. These findings, together with our above findings suggesting differential prognostic effects of PAR-1 and PAR-2 depending on ER status, motivated further analyses of potential interactions between these three factors. A Cox-model for DDFS with the three main effects for ER, PAR-1 and PAR-2, the three two-way interaction terms ER*PAR-1, ER*PAR-2 and PAR-1*PAR-2, and finally a term ER*PAR-1*PAR-2 for the three-way interaction, suggested a three-way interaction, which was almost significant (P = 0.070). To illustrate this interaction effect, prognosis in subgroups defined by PAR-1 and PAR-2 was studied separately for patients with ER-positive and ER-negative tumors (**Fig 3**). The HR for PAR-2 high *vs*. PAR-2 low in the PAR-1 high subgroup was 1.9 (95% CI: 0.31–11; P = 0.5) compared to HR = 12 (95% CI: 2.7–57; P = 0.001) in the PAR-1 low group. The DDFS in ER-positive/PAR-2 high/PAR-1 low and ER-positive/PAR-2 high/PAR-1 high was 62% and 90%, respectively (**Fig 3A**). The HR for PAR-1 high *vs*. PAR-1 low in the PAR-2 high subgroup was 4.2 (95% CI: 0.91–19; P = 0.065) compared to HR = 1.4 (95% CI: 0.28–6.9; P = 0.7) in the PAR-2 low group. The DDFS in ER-negative/PAR-1 high/PAR-2 low and ER-negative/PAR-1 high/PAR-2 high was 75% and 64%, respectively (**Fig 3B**). Although these subgroup analyses were clearly limited by reduction of sample size and should be interpreted with caution, the prognostic effect of PAR-2 in ER-positive patients appeared to be attenuated by concomitant high PAR-1 expression. On the contrary, the effect of PAR-1 in ER-negative tumors may be reinforced by concomitant high PAR-2 expression.

## Discussion

The major finding of the present study is that high PAR-2 expression strongly correlates with poor prognosis in a large patient subgroup, *i*.*e*. with luminal A like tumors [27], whereas PAR-1, on the contrary, appeared to be a negative prognostic factor specifically in the ER-negative subgroup. The classical view of ERα as an intracellular receptor that becomes activated and translocates to the nucleus only upon binding to its steroid hormone ligand has been abandoned through the elucidation of several mechanisms of nonclassical, estrogen-independent ER activation. These include post-translational ER modifications by *e*.*g*. phosphorylation at several positions dependent on receptor tyrosine kinases and G-protein coupled receptors and their downstream signaling molecules. This crosstalk should be of particular importance in the context of endocrine therapy resistance that may evolve as a result of estrogen-independent ER activation [28–32]. Interestingly, unrelated studies have shown that PI3K/AKT and ERK1/2 pathways are major downstream targets of PAR-2 activation [3], and that the same kinases can phosphorylate and activate ER independently of estrogen [27, 31]. Notably, several groups have shown that whereas PAR-1 transiently recruits β-arrestins, PAR-2 forms stable complexes with β-arrestins that function as a scaffold to promote ERK1/2 activation [15, 33–35]. Further, PAR-2 and ER signaling may merge at and synergize through common downstream signaling pathways, such as the MAPKs, or at the level of co-transcriptional regulation. ER can be recruited to transcriptional initiation sites other than estrogen responsive elements, which requires the association with other transcription factors [36–38] that may be connected with PAR-2 signaling. These potential signaling cross-talks between proteolytic activation of PARs and estrogen-dependent signaling through *e*.*g*. PI3K/AKT and MAPK pathways should be interesting avenues of future studies.

Our observation on the role of PAR-1 in ER-positive *vs*. ER-negative breast tumors should be discussed in the context of a previous study showing that tumors positive for ER and PAR-1 had a worse prognosis as compared with ER-positive tumors negative for PAR-1 [19]. The patient cohort in the previous study included pre- and postmenopausal patients (age range, 20–82) that were both lymph node-negative and–positive, whereas our study is based on a more homogenous cohort of mostly premenopausal (age range, 30–57), and lymph node-negative patients. Also, while the extent of adjuvant treatment (*e*.*g*. with antiestrogens) in the previous study is unknown, 87% of patients in the present study did not receive such treatment. Thus, differences in ER signaling status and available systemic estrogen levels between the respective patient cohorts may be a contributing factor to the discrepant results between studies.

Together, our results suggest that expression levels of PAR-1 and PAR-2 associate with breast cancer outcome in an ER-dependent manner. These observations motivate further mechanistic studies to unravel how the proteolytic activity of the tumor microenvironment and PAR activation may dictate ER-dependent signaling events during breast tumor development and metastasis.

## Supporting Information

S1 Fig Representative IHC stainings according to graded score for PAR-1 (upper panels) and PAR-2 (lower panels). (TIFF)

S2 Fig Cumulative incidence of breast cancer mortality in relation to PAR-2 (a) and PAR-1 (B) status. (TIF)

S3 Fig Cumulative incidence of breast cancer mortality in relation to ER and (a) PAR-2 or (b) PAR-1 status. (TIF)
