# Supplementary figures and images for: Protease Activated Receptors 1 and 2 Correlate Differently with Breast Cancer Aggressiveness Depending on Tumor ER Status
Source: PLoS One. 2015 Aug 5;10(8):e0134932. doi: 10.1371/journal.pone.0134932 (PMC4526525; doi:10.1371/journal.pone.0134932)

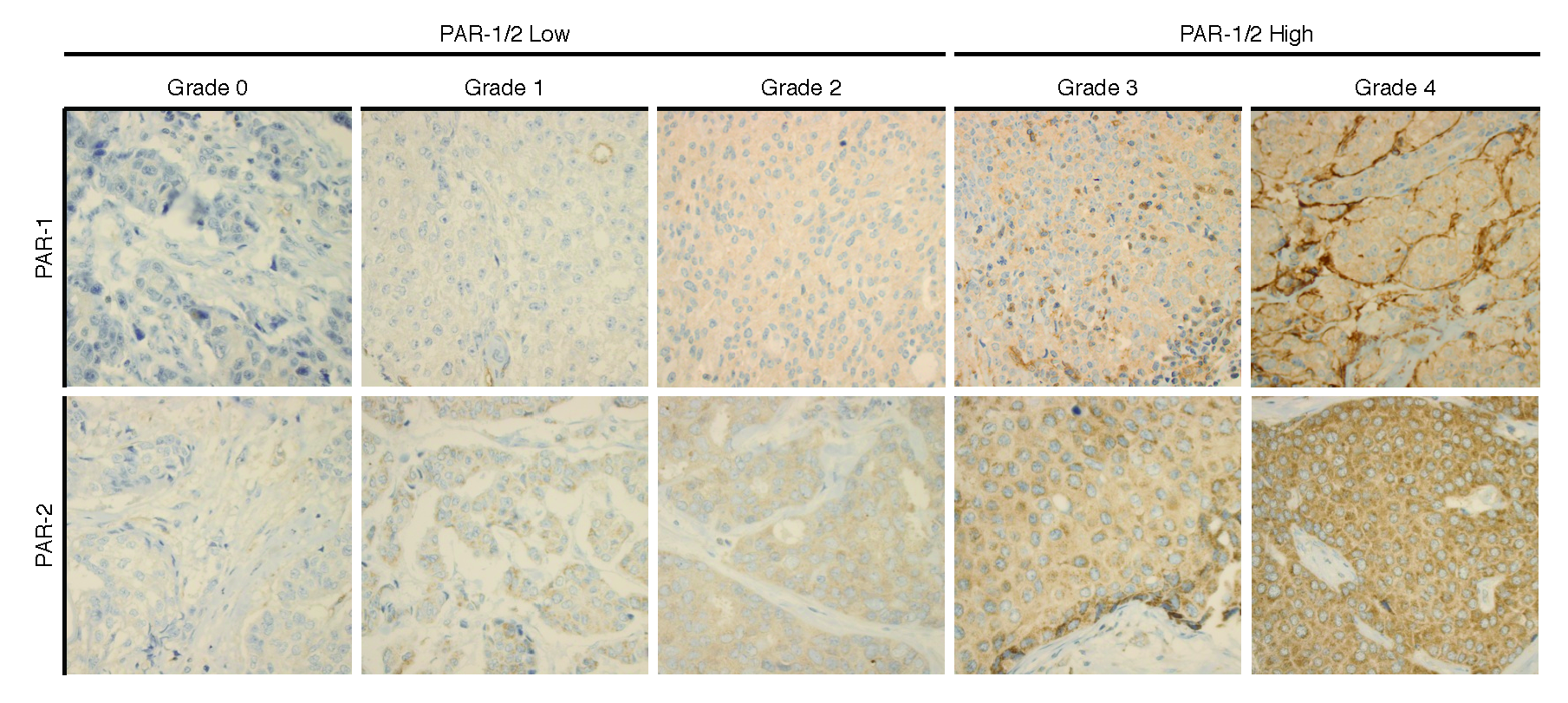

Supplement: S1 Fig — (TIFF) [file pone.0134932.s001.tiff]

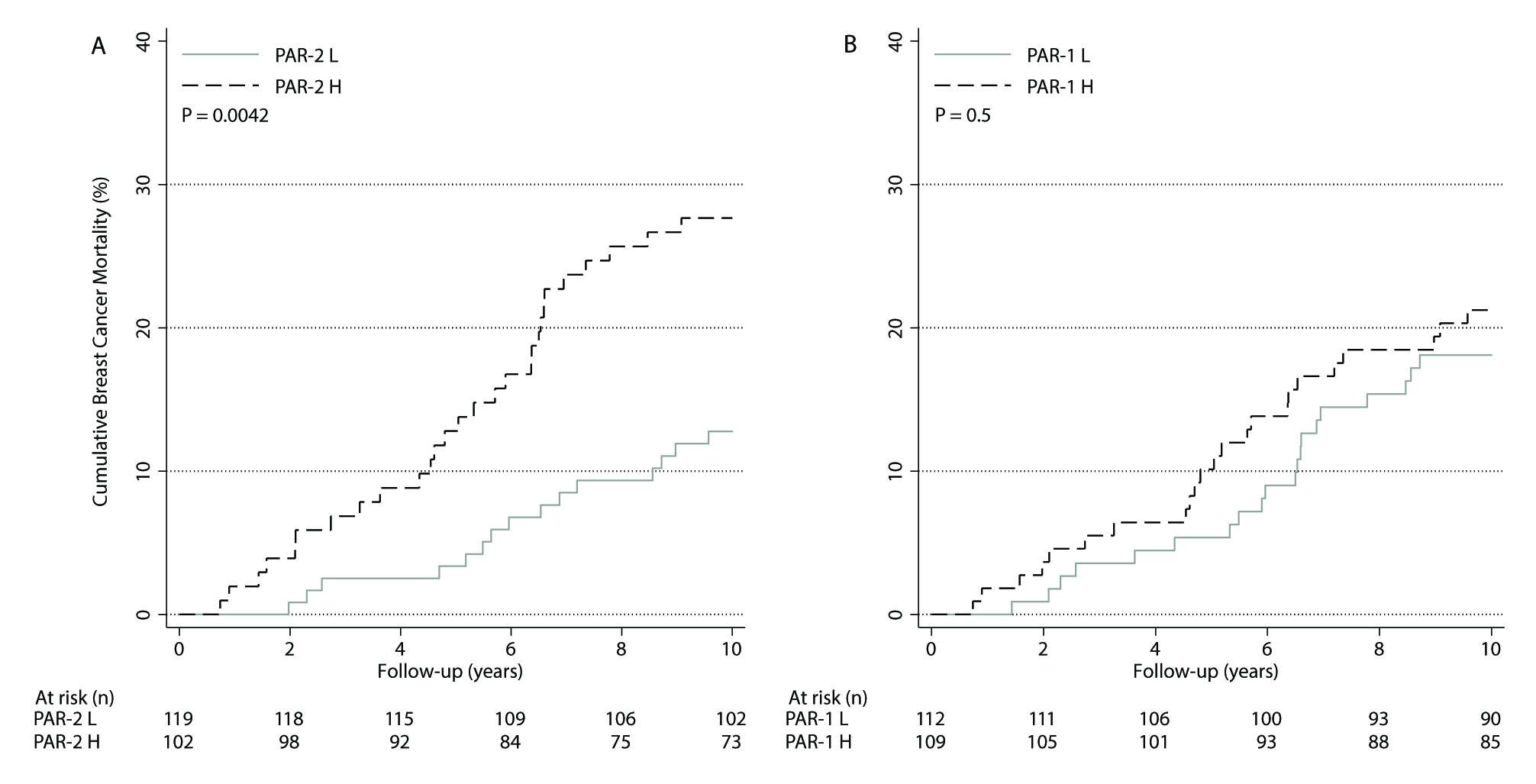

Supplement: S2 Fig — (TIF) [file pone.0134932.s002.tif]

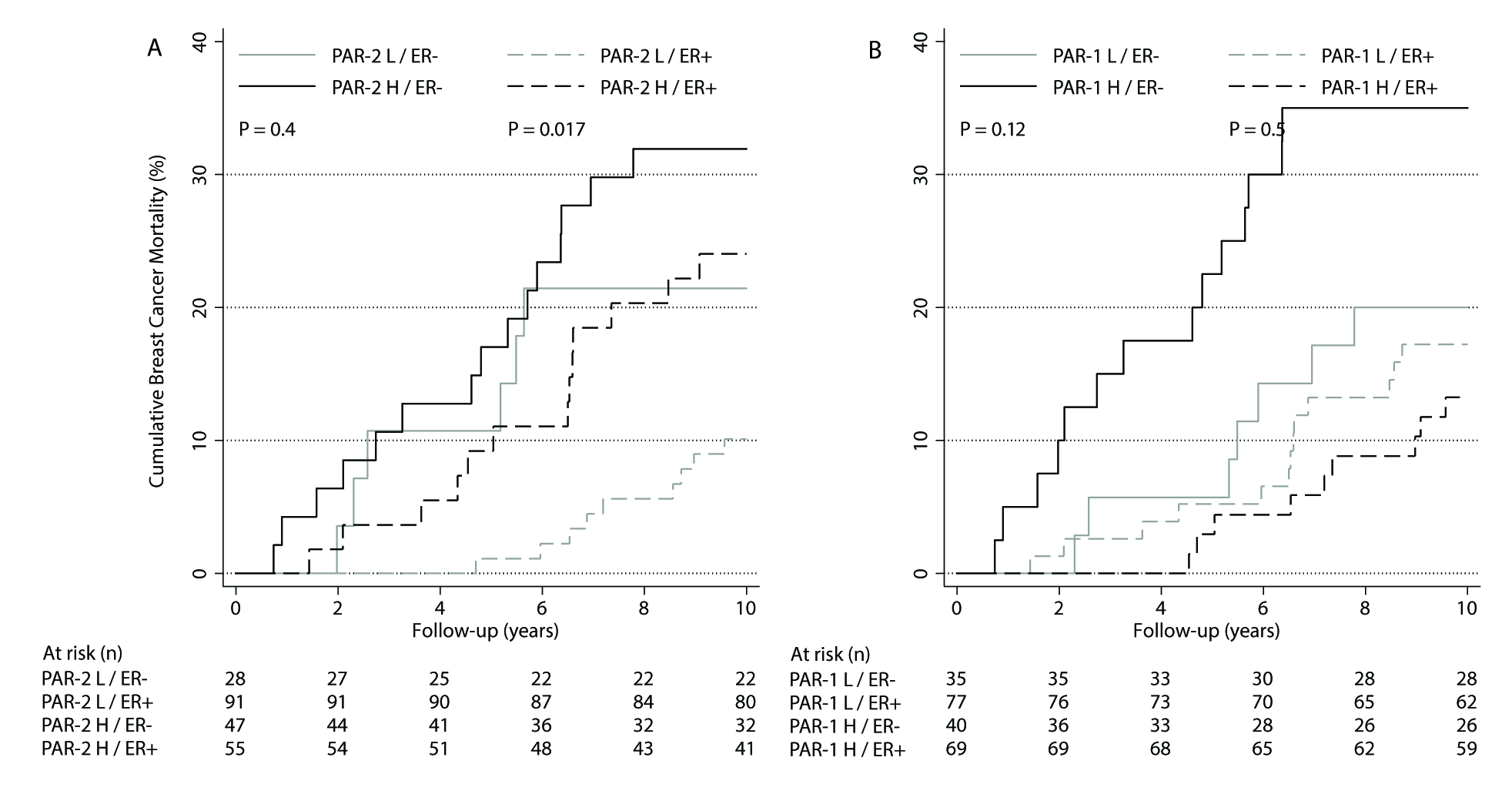

Supplement: S3 Fig — (TIF) [file pone.0134932.s003.tif]
